# Iris-Occlusion of XEN Gel Stent following Ab Externo Transconjunctival Implantation Technique

**DOI:** 10.1155/2021/2936047

**Published:** 2021-10-06

**Authors:** Sunil Ruparelia, Nir Shoham-Hazon

**Affiliations:** ^1^Faculty of Medicine, Dalhousie University, Halifax, Nova Scotia, Canada; ^2^Miramichi EyeNB Centre of Excellence, Miramichi, NB, Canada

## Abstract

The use of minimally invasive glaucoma surgery (MIGS) devices has become increasingly common for the management of elevated intraocular pressure (IOP) in the context of glaucoma. These technologies have traditionally been associated with fewer postoperative complications than conventional surgical techniques. However, we report on a rare case of transient XEN occlusion associated with pupil dilation following XEN gel stent implantation. This case highlights that in future XEN implantations, it may be preferable to position the XEN at a lesser angle to the iris to prevent such an occlusion. The use of different positionings of XEN is performed to optimize outcomes. However, it is highlighted that complications may arise in certain circumstances.

## 1. Introduction

Glaucomatous vision loss remains the leading cause of irreversible vision loss worldwide [1]. When topical therapy to manage intraocular pressure (IOP) fails, surgical intervention is indicated to prevent further progression of glaucomatous optic neuropathy. In recent years, minimally invasive glaucoma surgeries (MIGS) have become increasingly common in the management of uncontrolled glaucoma. Among these techniques is the XEN gel stent. The XEN gel stent is implanted beneath the conjunctiva, such that a shunt is created to drain aqueous humour from the anterior chamber of the eye into the subconjunctival space [2]. This is typically performed in an *ab interno* fashion but may also be done *ab externo* with or without dissection of the conjunctiva. These approaches have similar efficacies in terms of achieving target IOP and have similar safety profiles [3]. A major appeal of MIGS techniques is that they are typically associated with a lower complication rate than traditional surgical techniques [2]. However, we report on a rare case of transient iris-occlusion of the XEN gel stent 5 months following *ab externo* transconjunctival XEN gel stent implantation.

## 2. Case Presentation

A 56-year-old white male presented to the clinic for a 5-month postoperative follow-up appointment. The patient had received a superotemporal XEN gel stent implant in his left eye for management of his primary open-angle glaucoma (POAG). XEN implantation had been augmented with 0.2 mL of 0.3 mg/mL MMC, injected in an air-OVD fashion to create a pocket between the conjunctiva and Tenon's capsule.

At presentation, Snellen visual acuity was 20/60 in the left eye, and IOP was on target at 10 mmHg. However, following pupil dilation, an intraocular pressure (IOP) spike to 24 mmHg by Goldmann applanation was observed in the left eye. Other than the patient's existing diagnosis of POAG, no other ocular history or presenting symptoms were reported.

**Figure 1:**
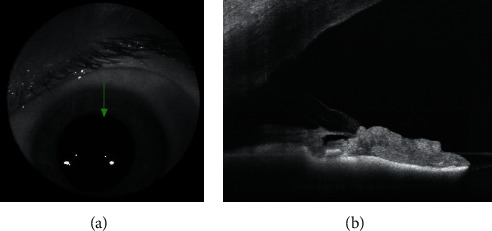
XEN stent placement (a) and anterior segment OCT (b) showing obstruction of XEN stent lumen after pupil dilation.

Prior to XEN gel stent surgery, pressure in the patient's left eye was 32 mmHg by Goldmann applanation on maximal tolerated medical therapy (MTMT). At that time, Snellen visual acuity in the left eye was 20/60. Following initial surgery, the patient had been discharged with a postoperative drop regimen including a quinolone antibiotic for 1 week and prednisolone 1% for 2 months postoperatively. Following the procedure, IOP had remained within a range of 5-10 mmHg measured at 1-month postoperative intervals without any IOP-lowering medications. After the IOP spike to 24 mmHg with dilation, multiple challenges with dilating drops demonstrated consistent rise in the left eye IOP to 24 mmHg to 30 mmHg during dilation. An anterior chamber OCT was performed and confirmed suspected XEN stent occlusion associated with pupil dilation (Figure 1). The patient was hesitant to undergo further intervention to prevent these events. As such, an observational management approach was taken in accordance with patient preferences.

## 3. Discussion

The XEN stent is one of the various microinvasive glaucoma surgery (MIGS) technologies that have been introduced in recent years. Since the FDA approval of XEN in 2016, its use has become increasingly common to manage patients with elevated IOP. A major appeal of the XEN implant is that it has been associated with fewer intra- and postoperative complications than traditional surgical techniques [2]. We describe the occurrence of a rare complication following XEN gel stent implantation.

Traditionally, the XEN is placed such that the device sits approximately 35 degrees under the conjunctiva and tenon [2]. However, further innovation with XEN implantation technique has led to variations in positioning of the device. In this case, XEN gel stent implantation was performed in a transconjunctival manner, with the air-OVD technique for tenon separation. The implant was positioned such that it was perpendicular rather than parallel to the iris in the anterior chamber. This implanted angle was sufficient to occlude the XEN upon pupil dilation.

Previous studies have outlined XEN occlusion by a variety of etiologies, including blood clots and fibrous scar tissue [4, 5]. However, literature regarding iris-occlusion of XEN gel stent is sparce. When faced with iris-occlusion of the XEN gel stent, there are several treatment options that may be considered. In our case, the patient was reluctant to receive further intervention, and we elected to observe the eye to determine whether further interventions would be required. Thus, we describe a conservative observational approach to iris occlusion of XEN gel stent. Several treatment modalities for iris-occluded XEN gel stent have also been described in the current literature. One case describing management of this complication reported reoccurrence of occlusion following surgical repositioning of XEN, but successful resolution of occlusion with combined treatment with argon laser peripheral iridoplasty and low energy neodymium-doped yttrium aluminum garnet laser [6]. Another report describes successful resolution of occlusion with argon laser peripheral iridoplasty alone [7].

The use of XEN gel stent has become increasingly common in practice. With continued innovation in implantation technique, it is important for care providers to have an understanding of the presenting symptoms, available investigations and management options to potential complications which may arise.

This case outlines a unique occurrence of XEN gel stent occlusion following pupillary dilation. In future XEN implantations, it may be advantageous to position the XEN at a lesser angle to the iris to prevent such an occlusion. This would require the surgeon to place the implant more anteriorly, aiming towards the cornea and parallel to the iris when deploying the device. Although innovation in technique with different positioning of XEN is performed with the intention of optimizing outcomes, we highlight that caution should be taken when innovating an already innovative technique.
